# On the transferability of pure electron correlation of quantum topologically defined molecular fragments at the CCSD(T) level

**DOI:** 10.1007/s11224-026-02730-8

**Published:** 2026-04-21

**Authors:** Mark A. Vincent, Paul L. A. Popelier

**Affiliations:** Department of Chemistry, The University of Manchester, Manchester, M13 9PL Great Britain

**Keywords:** QTAIM, CCSD (T), Electron correlation, Interacting Quantum Atoms (IQA), Dispersion, Two-particle density-matrix

## Abstract

The Interacting Quantum Atoms (IQA) method offers a route to calculate, directly from the two-particle-density-matrix, electron correlation energies for a quantum topological atom interacting with itself and with other atoms in a given system. Here, we corroborate the energetic transferability, at CCSD(T) level, of a variety of functional groups as they appear in both small organic molecules and water-containing complexes: methyl, amine, hydroxyl, water, carbonyl, carboxyl, amide, methylene and triple bonds. The dependence of these atomic correlation energies upon variation of a dihedral angle is very small (0.1 kJ/mol), and that upon variation in bond length and angle reasonably small (units of kJ/mol for molecular dynamics-induced changes and tens of kJ/mol for extreme, manual changes). Overall, these findings give the green light for the machine learning of these physically-informed atomic properties. The beneficiary is the force field FFLUX, which will then be able to make predictions on dispersion effects, totally in line with the other types of IQA energy contributions that underpin it. Hence the familiar dispersion schemes that are typically bolted on become unnecessary.

## Introduction

A major activity of computational chemistry is overcoming the huge challenge of fast and accurate prediction of the properties of systems consisting of many thousands of atoms, ideally over a long (atomic) time span. Frustratingly, overcoming this challenge from first principles remains unmet, at least for any practical purposes, in spite of the breathtaking development of sophisticated algorithms and the increasing adoption of post-CPU hardware. While this unmet challenge persists today, already many decades ago it prompted a very different approach: force fields.

Classical force fields depart from any physical (i.e. quantum) insight into why atoms behave the way they do. For example, if the energy of two bonded atoms increases when their internuclear distance decreases, then the force field does not “know” or explain *why* this is so. Instead, it just captures this energetic behaviour by a simple mathematical expression containing parameters. The latter need to acquire trustworthy values, which for a complex system soon turns out to be problematic, as the community learnt the hard way, over many years. In fact, an inconvenient truth is that decades of force field design have deprived a huge number of users of accurate predictions that experimental (bio)chemists can take seriously. Often minor variations in force fields, either in the shape of their expression or in the values of the parameters, continues to lead to huge disparities in their predictions of the structure and dynamics of, for example, oligopeptides in aqueous solution [1]. However, the advent of machine learning (ML) used in the design of interatomic potentials has helped make force fields more realistic. But then again, ML itself introduced challenges of its own, a well-known one being the quality of the training used. Its size, relevance and diversity all matter.

However, in this work we focus on another challenge: atomic transferability. This is the key concept behind any force field, whether classical or ML-based. Indeed, the chemical nature itself of ambient matter divulges local patterns of near constancy. In other words, atoms or small sets thereof, reveal values for physical properties (e.g. charge, dipole moment or energy) that are very similar, regardless of the composition of the larger system that these atoms belong to. Thanks to this remarkable locality force fields make sense; without it they would be a dead end because each of the currently known ~ 200 million proteins, for example, would need its very own set of parametric values, or even worse, its own mathematical expressions.

Over many years now, we have systematically and carefully constructed a novel type of force field called FFLUX [2–4] although a recently emerged development community would call it a machine-learned interatomic potential (MLIP). FFLUX is completely detached from any existing classical force field, which amplified its construction time. However, more importantly, this detachment frees it from any of the inherent limitations of such force fields.

FFLUX is built on four cornerstones. Firstly, it adopts the Quantum Theory of Atoms in Molecules (QTAIM) [5–7] to define its atoms and their properties. Secondly, FFLUX takes advantage of the accuracy of high-rank multipolar electrostatics for the long-range interaction between atoms, while also benefitting from the rigour of multipolar Ewald summation [8]– [9]. Thirdly, the short-range potential energy is calculated using a method called Interacting Quantum Atoms (IQA) [10], which was inspired by our calculation [11] of Coulomb energy within an atom and between (different) atoms in non-equilibrium systems, made possible by 6D integration over the respective volumes of two quantum atoms. We note that IQA and QTAIM both belong to Quantum Chemical Topology [12]– [13], a methodology that harnesses the mathematical langue of dynamical systems to quantum mechanical densities. Fourthly and finally, the ML technique Gaussian Process Regression [14], formerly known as Kriging [15], captures how atomic properties change in response to the changing geometry of their environment. When this property is the atomic dipole moment, then (dipolar) polarisation is predicted by the ML. Thus, FFLUX is a polarisable force field. Furthermore, if the atomic property is atomic charge then it is charge transfer that is being modelled, a notoriously difficult feat in the context of Hilbert-space-based energy decomposition analyses (EDA). However, because IQA is a real-space energy partitioning (that does not follow the principles of perturbation theory) it does not suffer from the conceptual and numerical difficulties that the former EDAs have.

In fact, it turns out that IQA offers a remarkably stable and minimal set of energies none of which depends on reference states (and the issues they cause). Table 1 shows the four basic types of energy that IQA provides in terms of their clear chemical narrative.

**Table 1:** The physical energy types associated with IQA and its chemical interpretation

Energy type	Physical Foundation	Physicochemical	Chemical concepts
1	Kinetic energy, intra-atomic electron-electron and electron–nucleus potential energy	Steric energy	Steric hindrance
2	Interatomic Coulomb	Electrostatics	Polarity, ionicity
3	Interatomic exchange energy	Bonding Pauli repulsion	Bond order, (hyper)conjugation
4	Interatomic electron correlation energy	Part of van der Waals interaction	dispersion

An important question is now whether atomic electron correlation shows transferability, in particular, when generated at the gold standard of CCSD(T). This paper investigates this question based on dozens of simple organic molecules covering functional groups such as methyl, amino or hydroxyl moieties. An affirmative answer is crucial for FFLUX because then we can confirm that it is indeed trained on chemically meaningful information. This, in turn, means that the ever-so-difficult-to-model dispersion energy will exist in FFLUX totally consistent with IQA, which provides the other types of energy. We already obtained proof-of-concept [16] a while ago that Gaussian Process Regression can predict electron correlation energies (admittedly for small systems) within 0.5 kJ/mol with very small training sets. Very recently, we confirmed and expanded [17] this success for water trimers allowing for an error up to 1.3 kJ/mol as obtained by delta-learning, which offers an enormous computational speed-up. Hence, there will be no need to import external and ad hoc dispersion corrections, which often introduce problematic damping functions (for example ref [18]). Following this strategy, the groundwork is currently being laid for FFLUX to model (ambient) matter in a future-proof manner.

## Theory and computational details

### Background

We have explained the theory behind our approach elsewhere [19]– [20] but briefly summarise it here. The electron-electron potential energy $\:{V}_{ee}^{AB}$ is the most complicated contribution in the IQA approach, more complicated than the atomic kinetic energy, the electron-nucleus attractive potential energy or the nucleus-nucleus repulsive potential of course. Mathematically, it is defined by Eq. 1,

1 $$\begin{aligned}V_{ee}^{AB} =& \sum_{j=1}^{N_{basis}} \sum_{k=1}^{j} K_{jk} \sum_{l=1}^{N_{basis}} \sum_{m=1}^{l} K_{lm} d_{jklm} \int_{\Omega_A} d\mathbf{r}_1 G_{jk}(\mathbf{r}_1 - \mathbf{R}_{jk}) \\ &\int_{\Omega_B} d\mathbf{r}_2 \frac{1}{r_{12}} G_{lm}(\mathbf{r}_2 - \mathbf{R}_{lm})\end{aligned}$$

where $N_{basis}$ is the number of primitive Gaussian basis functions, $G_{jk}$ and $G_{lm}$ each are the respective product functions, each of two Gaussian indexed basis functions, Ω is the volume of an atom and each element of the inevitably huge 2PDM is designated by $d_{jklm}$ *.* Note that $V^{AB}$ can become the electron correlation energy $\:{V}_{ee,corr}^{AB}$ if *d*_*jklm*_ is restricted to the pure electron correlation part of the 2PDM. This specification can be achieved by subtracting from it the Hartree-Fock (HF) Coulomb and exchange part of the 2PDM. However, we can go further and subtract the approximate Müller 2PDM from the 2PDM. The Müller approach [21] is a 1-electron-based estimate to successfully approximate the 2PDM, an approach that has been used many times by ourselves and others. The Müller method captures most of the electron correlation well, such that the pure 2-electron-based electron correlation that it leaves behind is a small residue [22]. Although it is small, it is mandatory for it to be included in the quest for ultimate accuracy. Finally, we note that each subscript *j*, *k*,* l* and *m* in Eq. 1 denotes a basis function while *K* (of course not to be confused with the index *k*) is a constant resulting from having multiplied two Gaussian basis functions.

We point out that in Eq. 1 there is no reference to which wavefunction the 2PDM came from. Put differently, the equation is universally valid for any 2PDM. However, for convenience we introduce a notation that specifies the source of the 2PDM at hand: 2PDM-M/CCSD(T) where *M* refers to the Müller approximation. In other words, from the full CCSD(T) 2PDM the Müller-approximated 2PDM has been removed. The 2PDM or 2PDM-M matrices have the same number of matrix elements, whichever level of theory (e.g. MP2, MP3, MP4SDQ, CCSD or CCSD(T)) was used to generate them. It is the values of these matrix elements that vary with the level of theory.

We note that the “correlation energy” definition used in the paper is non-standard because the “normal” electron correlation energy arises from subtracting the HF energy from the post-HF method’s energy (e.g. CCSD(T)). Because the HF energy is always higher than the correlated CCSD(T) energy the electron correlation energy is always negative for multi-electron systems. However, the electron correlation energy definition used in this paper is the difference between the CCSD(T) energy and the energy calculated using the Müller 2PDM. The Müller approximation typically overestimates the magnitude of the electron correlation energy, (i.e. it is too negative), thus, the “correlation energy” definition used in the paper leads to positive values, which comes across as unusual when used when working with the standard definition of correlation energy.

From hereon, the $\:{V}_{ee}^{AB}$energy will be referred to as the *ee* energy (A-B energy for short), as it describes the interaction energy between a given atom *A* and a single atom *B*. However, it is possible to sum over the atoms *B*, which include *A* itself, to give an integral over all space. In other words, the $\:{V}_{ee}^{AB}$ energies summed in this way yield the energy of *A* with the rest of the molecule (including itself). Because all the atoms in a molecule (i.e. the full system) are covering all space this energy derives from an integral over all space. We can actually replace this sum by an analytic integral to yield the two-electron energy of *A* in a molecule. This approach is what we call the A-A’ method. Normally the notation A’ refers to the rest of the molecule, that is, the molecule minus the *A* atom. Hence, the interaction between atom *A* and the whole molecule would be denoted A-(A + A’), where it is clear that *A* interacts with itself. However, for compactness we use the notation A-A’ to refer to atom *A* interacting with the rest of the molecule, including itself. An alternative name for this method, namely ESP, is inspired by the way an A-A’ energy is calculated. The ESP name arises from the fact that this analytic integration is in fact equivalent to an **e**lectro**s**tatic **p**otential integral (or ESP integral in short). Note that in the ESP approach only one of the two integrations in Eq. 1 is now analytic. In other words, Eq. 1 shows a double integration, one over atom *A* and one over atom *B*; by summing over *B* we only remove the numerical integration over *B* and turn it into an analytic one while the numerical integration over atom *A* remains. As we noted previously, replacing a numerical integration by an analytic one, reduces the numerical error of the *two-electron* energies.

However, we still need to carry out one numerical integration, which involves the installation of a quadrature grid. The angular grid is efficiently constructed following Lebedev’s method resulting in 350 points judiciously positioned on a sphere surrounding the nucleus. This grid is coded by the number “15” which appears first in the *(p*,* q)* designation of the grid, as *p*. The number *q* represents the number of radial grid points. Hence, the number quadrature points within the so-called β sphere is 350 × 15 = 5,250. The atomic volume outside the β sphere is also endowed with the same number of points, which brings the total number to 10,500 points. This is a minimum number of integration points because additional grid points can be added when complicated shaped atomic volumes are encountered. Thus, we sometimes employed a grid referred to as (20,20), which has 23,600 grid points for the combined β and outer spheres.

### Software

**Fig. 1:**
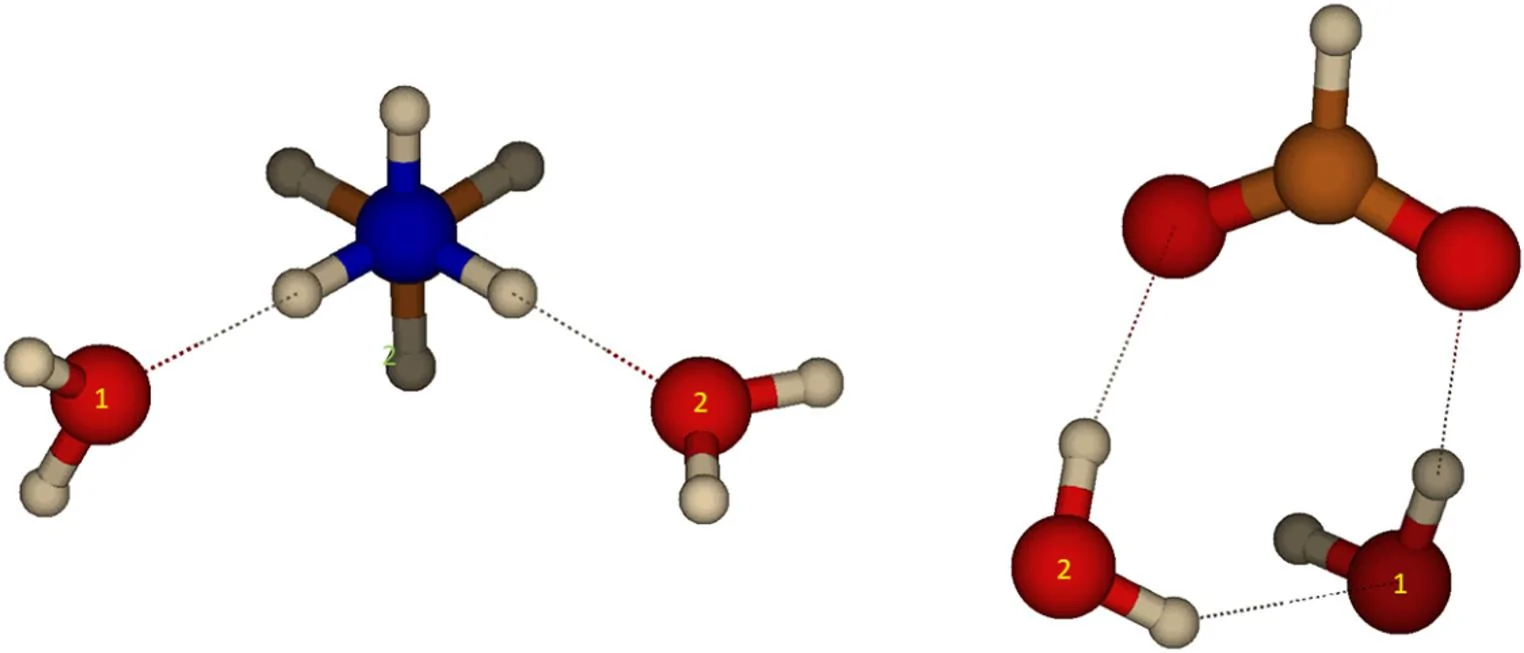
The solvating water molecules of protonated methylamine and the formate ion

We used the program GAUSSIAN09 [23] to obtain most local-energy-minimum geometries of the molecules and ions studied here, employing the CCSD/uncontracted 6–31 + G(d, p) level of theory, with the exceptions specified later. These geometries were then passed to the program [24] PySCF where the one-particle density matrix (1PDM) and 2PDM was generated via the CCSD(T)/uncontracted 6–31 + G(d, p) method, along with the ESP integrals and a ‘WFN’ file. In addition, the 1PDM was diagonalised to yield the natural orbitals (eigenvectors) and occupation numbers (the eigenvalues). These eigenvectors and eigenvalues were then passed to another in-house code along with the 2PDM, to generate the required form of the 2PDM (i.e. 2PDM-M/CCSD(T)). The ‘WFN’ file passes the wavefunction information (geometry, basis set, occupation numbers and natural orbitals) to our in-house program MORFI. Thus, MORFI receives the WFN file and the 2PDM-M/CCSD(T) along with the ESP integrals. MORFI then produces the required A-A’ energies, generally [22] using the (15,15) grid. We have studied a series of hydrocarbons (CH_4_ to C_8_H_18_). However, because of the size of the later members of this series, we employed the computationally cheaper CCSD method, again with the uncontracted-6–31 + G(d, p) (i.e. 2PDM-M/CCSD/uncontracted 6–31 + G(d, p)). This yields the largest 2PDM having a size of ~ 428 Gigabyte. The geometries were determined at the MP2(full)/uncontracted 6–31 + G(d, p) level again using GAUSSIAN09. The statistics and concomitant plots were produced by Excel, where the AVERAGE and STDEVP built in functions were employed. The quantum topological program [25] AIMAll was used to calculate the atomic energies and charge of atoms in triple-bonded systems. Fig. 1 was generated via MOLDEN [26] and Powerpoint.

## Results and discussion

### The methyl group

Table 2 presents the atomic energies of the carbon and the three hydrogen atoms of various methyl groups arising from our study of a variety of more than 50 compounds containing these CH_3_ groups. In addition, the five columns on the right of Table 2 gives the differences between the energy of the methyl group in staggered ethane and that of various methyl groups. Ethane is the obvious reference for a “pure” methyl group because this molecule simply consists of two methyls attached to each other. From the listed energy differences one can determine to what extent a standard methyl group exists. In other words, does a pattern reveals itself in the correlation energy of methyl groups in a wide variety of bonding situations?

**Table 2:** The *A-A’* atomic energies (kJ/mol) of various Methyl groups (columns 2 to 6) in different molecules calculated with the (15,15) grid. Columns 7 to 11 list the energy differences with reference molecule ethane. The H1 atom is the one lying in a plane, if such a plane exists. The energy values for the other two hydrogens have been averaged if there is global symmetry in the compound. Two different total average values are given in the last two rows, with the standard deviation in brackets. Method: 2PDM-M/CCSD(T)/uncontracted 6–31 + G(d, p). The sum in the utmost right column is a sum of the absolute differences. If more than one Methyl group is present in a molecule, then the Methyl referred to is marked in bold

Molecule	C of CH_3_	H1	H2	H3	sum	C of CH_3_	H1	H2	H3	sum
CH_4_	74.2	26.6	26.6	26.6	154.1	−13.0	−3.2	−3.2	−3.2	22.7
*C*_*2*_*H*_*6*_ *(reference molecule)*	*87.2*	*29.9*	*29.9*	*29.9*	*176.8*	*0.0*	*0.0*	*0.0*	*0.0*	*0.0*
C_3_H_8_ (asym)^a^	89.9	31.1	30.3	30.3	181.5	2.7	1.2	0.4	0.4	4.8
C_3_H_8_ (sym)^a^	89.8	31.1	30.3	30.3	181.5	2.6	1.2	0.4	0.4	4.7
C_4_H_10_ (*n*-butane)	90.7	31.6	30.6	30.6	183.6	3.5	1.8	0.7	0.7	6.8
CH_3_NH_2_	90.6	30.7	30.0	30.0	181.3	3.5	0.9	0.1	0.1	4.5
C_2_H_5_NH_2_	90.5	31.1	30.3	30.5	182.3	3.3	1.3	0.4	0.6	5.6
C_3_H_7_NH_2_	91.4	31.6	30.8	30.7	184.5	4.2	1.8	0.9	0.9	7.8
*iso*-C_3_H_7_NH_2_	92.4	31.5	31.5	30.6	186.0	5.3	1.7	1.6	0.7	9.2
CH_3_SH	93.0	30.5	30.9	30.9	185.4	5.9	0.6	1.0	1.0	8.6
CH_3_SCH_3_	95.2	31.6	31.2	31.2	189.2	8.0	1.7	1.4	1.4	12.5
CH_3_CO_2_H	96.1	30.9	31.7	31.7	190.4	8.9	1.0	1.8	1.8	13.6
CH_3_OH	92.9	29.5	30.3	30.3	183.0	5.7	−0.4	0.5	0.5	7.0
C_2_H_5_OH	89.8	30.8	30.1	30.1	180.8	2.7	0.9	0.2	0.2	4.0
C_3_H_7_OH	91.0	31.4	30.7	30.7	183.7	3.8	1.6	0.8	0.8	6.9
*iso*-C_3_H_7_OH	92.8	31.3	31.3	30.5	186.0	5.7	1.5	1.5	0.7	9.3
CH_3_CONH_2_	97.2	30.9	32.0	32.0	192.2	10.1	1.0	2.2	2.2	15.4
CH_3_CHO	95.9	30.1	31.6	31.6	189.2	8.8	0.2	1.7	1.7	12.4
CH_3_CH_2_CHO	91.5	31.7	30.8	30.8	184.9	4.3	1.8	1.0	1.0	8.1
*n*-C_3_H_7_CHO	92.2	31.9	31.1	31.1	186.4	5.0	2.1	1.2	1.2	9.6
*iso*-**CH**_**3**_C(H)(CH_3_)CHO *cis* to O	93.4	32.0	31.2	31.7	188.3	6.2	2.1	1.3	1.9	11.6
*iso*-CH_3_C(H)(**CH**_**3**_)CHO *gauche* to O	94.3	32.0	31.3	31.9	189.5	7.1	2.2	1.4	2.0	12.8
CH_3_COCH_3_	97.5	31.0	31.9	31.9	192.2	10.3	1.1	2.0	2.0	15.4
**CH**_**3**_COCH_2_CH_3_	98.3	31.6	31.9	32.4	194.2	11.2	1.7	2.0	2.5	17.4
CH_3_COCH_2_**CH**_**3**_	92.4	32.1	31.0	31.1	186.7	5.2	2.2	1.2	1.3	9.9
CH_3_NH_3_^+^	89.4	27.9	27.9	27.9	173.1	2.2	−2.0	−2.0	−2.0	8.1
CH_3_NH_3_^+^.H_2_O	89.4	28.7	28.4	28.4	174.8	2.2	−1.1	−1.5	−1.5	6.3
CH_3_NH_3_^+^.(H_2_O)_2_	89.3	28.6	28.9	28.9	175.6	2.1	−1.3	−1.0	−1.0	5.4
CH_3_OCH_3_	95.8	30.9	30.9	30.9	188.4	8.7	1.0	1.0	1.0	11.7
CH_3_OCH_2_**CH**_**3**_	90.9	31.3	30.5	30.5	183.1	3.7	1.4	0.6	0.6	6.3
**CH**_**3**_OCH_2_CH_3_	97.1	31.5	31.3	31.3	191.2	9.9	1.6	1.4	1.4	14.4
(CH_3_-NH)C(NH_2_)NH_2_^+^	94.3	31.2	30.8	30.7	186.9	7.1	1.3	0.9	0.8	10.2
**CH**_**3**_CON(H)CH_3_	98.0	31.3	32.4	32.4	194.1	7.8	2.0	1.7	1.7	13.4
CH_3_CON(H)**CH**_**3**_	95.0	31.9	31.6	31.6	190.1	10.9	1.4	2.5	2.5	17.3
C_2_H_5_CO_2_^−^	96.2	33.9	32.3	31.6	194.0	9.1	4.0	2.4	1.7	17.2
C_2_H_5_CO_2_H	92.1	31.9	30.8	30.8	185.7	5.0	2.0	0.9	0.9	8.9
**CH**_**3**_(H)C = C(CH_3_)_2_	94.6	32.1	32.2	32.2	191.0	9.2	1.9	2.8	2.8	16.7
CH_3_(H)C = C(**CH**_**3**_)_2_ *trans*	96.4	31.8	32.6	32.6	193.4	8.6	1.5	2.6	2.6	15.5
CH_3_(H)C = C(**CH**_**3**_)_2_ *cis*	95.8	31.4	32.5	32.5	192.2	7.5	2.2	2.3	2.3	14.3
C_2_H_5_(H)C = CH_2_	92.5	32.1	31.0	31.1	186.7	5.4	2.2	1.2	1.2	9.9
(CH_3_)_2_C=CH_2_	95.2	31.5	32.1	32.1	191.0	8.0	1.7	2.3	2.3	14.2
CH_3_(H)C = C(H)CH_3_ *trans*	94.2	30.9	32.3	32.3	189.8	7.1	1.1	2.5	2.5	13.1
CH_3_(H)C = C(H)CH_3_ *cis*	94.5	30.5	32.4	32.4	189.7	7.3	0.6	2.5	2.5	12.9
CH_3_(H)C = CH_2_	97.0	32.2	32.2	32.2	193.4	9.8	2.3	2.3	2.3	16.7
CH_3_C ≡ CCH_3_	96.9	32.4	32.4	32.4	194.1	9.7	2.5	2.5	2.5	17.4
**CH**_**3**_C ≡ CCH_2_CH_3_	97.5	32.7	32.6	32.6	195.5	10.4	2.8	2.8	2.8	18.7
CH_3_C ≡ CCH_2_**CH**_**3**_	93.0	32.5	31.2	31.2	187.8	5.9	2.6	1.3	1.3	11.1
CH_3_CH_2_C ≡ CH	93.2	32.3	31.1	31.1	187.7	6.1	2.4	1.2	1.2	11.0
CH_3_CH_2_CH_2_C ≡ CH	92.7	32.3	31.3	30.9	187.2	5.5	2.5	1.4	1.0	10.4
CH_3_-C(H) = NH	94.4	31.1	31.7	31.7	188.9	7.3	1.3	1.8	1.8	12.2
CH_3_CH_2_C(H) = NH	93.2	32.1	31.2	31.2	187.7	6.1	2.2	1.3	1.3	10.9
CH_3_CH_2_CH_2_C(H) = NH	92.6	32.3	31.2	31.2	187.3	5.5	2.4	1.3	1.3	10.6
(**CH**_**3**_)_2_C=NH *trans*	96.2	31.4	32.4	32.4	192.4	9.1	1.5	2.5	2.5	15.6
(**CH**_**3**_)_2_C=NH *cis*	97.0	31.5	32.4	32.4	193.3	9.8	1.6	2.5	2.5	16.5
**CH**_**3**_C(H) = NCH_3_	95.3	31.7	32.1	32.1	191.3	8.1	1.8	2.3	2.3	14.5
H_3_C(H) = N**CH**_**3**_	97.9	32.1	32.3	32.3	194.7	10.8	2.3	2.4	2.4	17.9
H_2_C = NCH_3_	97.5	31.5	32.0	32.0	193.0	10.3	1.7	2.1	2.1	16.2
**CH**_**3**_-C(H) = NCH_2_CH_3_	95.7	31.0	32.8	32.8	192.2	8.5	1.1	2.9	2.9	15.5
CH_3_-C(H) = NCH_2_**CH**_**3**_	93.4	32.5	31.4	31.3	188.7	6.3	2.7	1.6	1.4	11.9
**CH**_**3**_(C_2_H_5_)C = NH	98.1	32.2	32.3	32.3	194.9	11.0	2.3	2.5	2.4	18.2
CH_3_(CH_2_**CH**_**3**_)C = NH	106.0	32.4	31.3	31.0	200.8	18.9	2.5	1.5	1.2	24.0
Average (SD) include CH_4_	93.7 (4.0)	31.3 (1.2)	31.2 (1.2)	31.2 (1.2)	187.5 (6.9)	6.6 (4.0)	1.4 (1.2)	1.3 (1.2)	1.3 (1.2)	11.9 (4.7)
Average (SD) exclude CH_4_	94.1 (3.1)	31.4 (1.0)	31.3 (1.0)	31.3 (1.0)	188.0 (5.4)	6.9 (3.1)	1.5 (1.0)	1.4 (1.0)	1.4 (1.0)	11.7 (4.6)

^a^The symmetric form of C_3_H_8_refers to the conformation with (global) C_2v_symmetry with the mirror plane containing the 3 carbons. The asymmetric form lacks any symmetry and is hence the point group is C_1_.

The column marked “sum” in the middle of Table 2 reports the sum of four energies: that of the methyl carbon and the three hydrogens bonded to it. The average correlation energy for a methyl group is 187.5 kJ/mol with a standard deviation (SD) of 6.9 kJ/mol. Because the SD amounts to only 4% of the average energy, the energy distribution can be considered as fairly narrow. However, with a minimum energy of 154.1 kJ/mol (methane) and a maximum energy of 200.8 kJ/mol (butane-2-imine, CH_3_(CH_2_**CH**_**3**_)C = NH, relevant methyl in bold) the range is 46.7 kJ/mol. But then again, the minimum energy is almost 5 SDs away from the average and the maximum energy almost 2 SDs. The penultimate smallest energy is that of methyl in CH_3_NH_3_^+^ with a value of 173.1 kJ/mol, which is only 2SDs away from the average. Hence, only methane is a strong outlier, and its justified omission triggers a recalculation of the average, resulting in 188.0 kJ/mol. The new SD then becomes 5.4 kJ/mol (2.8% of the average), which is about a quarter smaller compared to the old SD. Remarkably, the respective SDs for the carbon and the three hydrogens each amount to ~ 3% of their respective averages. Hence, the narrowness of the methyl-energy distribution is also reflected in that of the carbon and that of the hydrogens; there are no cancellation or reinforcement effects.

It is not the purpose of this paper to comment on all the subtle effects that can be gleaned from the more than 50 entries in Table 2, or from similar Tables covering other functional groups. Yet, there are some observations that are worth mentioning. Firstly, on average, the correlation energy of the whole methyl group is split half-way between the carbon on one hand and the three hydrogens on the other. Moreover, although the energies between the individual hydrogens can differ by ~ 1–2 kJ/mol, on average, however, they are identical (within 0.1 kJ/mol). Secondly, the deprotonation of propanoic acid has a large effect on the terminal methyl group, unusually increasing its correlation energy by ~ 8 kJ/mol. Thirdly, the presence of a C = O group (whether by itself as carbonyl, or in an amide or aldehyde group) when bonded right next to the methyl’s carbon, has a relatively large effect, increasing its correlation energy by 12–17 kJ/mol. Fourthly and finally, the immediate vicinity of a double or triple bond is also disruptive, raising its energy by ~ 15 kJ/mol or more.

### The effect on the Methyl group of chain length of aliphatic hydrocarbons

It is interesting to investigate if the *ee* energy of the terminal methyl asymptotically converges to a particular value with increasing chain length. Unfortunately, for CCSD(T) the sequence is limited to only four hydrocarbons, from methane to butane, generating the sequence 154.1, 176.8, 181.5 and 183.6 kJ/mol, respectively, each time for the whole methyl group. The corresponding sequence of consecutive differences (i.e. ethane minus methane, propane minus ethane, …) is 22.7, 4.7 and 2.1 kJ/mol, which is too short to confirm the expected convergence. However, the reduced computational cost of the 2PDM-M/CCSD method, again in combination with the uncontracted 6–31 + G(d, p) basis set, enables doubling the chain length to octane or C_8_H_18_.

Table 3 lists the results for the series CH_4_ to C_8_H_18_ involving geometries obtained at the MP2(full)/uncontracted 6–31 + G(d, p) level, for all molecules. A glance at the second column conveys that the energies of the CH_3_ group have converged by about C_5_H_12_, at a threshold of 0.1 kJ/mol. The energies of CH_2_ groups also show convergence but at a point in the homologous series that depends on the position of the CH_2_ within the molecule. For example, the CH_2_ right next to the terminal CH_3_ converges at heptane, while the next CH_2_ (lying more centrally in the molecule) converges only at octane. In other words, the more central the CH_2_, the further down the series that it converges. Finally, we note that the energy of the methyl group is 1 kJ/mol to almost 6 kJ/mol higher at CCSD(T) level compared to CCSD, in going from methane to butane.

**Table 3:** The *A-A’* energies of CH_3_ and various CH_2_ groups in the straight-chain homologous series CH_4_ to C_8_H_18_. Units: kJ/mol. Method: 2PDM-M/CCSD/uncontracted 6–31 + G(d, p). Grid is (15,15)

Molecule	CH_3_	CH_2_(-CH_3_)^a^	CH_2_(-CH_2_-CH_3_)	CH_2_(-CH_2_-CH_2_-CH_3_)
CH_4_	153.2	-	-	-
C_2_H_6_	173.6	-	-	-
C_3_H_8_	177.0	163.4	-	-
C_4_H_10_	178.0	165.2	-	-
C_5_H_12_	178.4	166.0	167.4	-
C_6_H_14_	178.5	166.3	168.2	-
C_7_H_16_	178.5	166.4	168.5	169.2
C_8_H_18_	178.6	166.4	168.5	169.1

^a^ We designate a group by referring to the smallest chain that it belongs to. For example, in C_4_H_10_ there is a “CH_2_(-CH_2_-CH_3_)” methylene group but this CH_2_ is also the methylene that occurs in CH_2_(-CH_3_). Because the latter chain is smaller it is the only reference point giving a non-empty entry in the table

### The amine group

Table 4 shows the results for the now smaller set (about a dozen) of molecules containing NH_2_ groups. Both hydrogens show a distribution of electron correlation energies that is somewhat broader than that of the hydrogens in the methyl, with a SD that is now 6% *versus* 4% before. Similarly to methane, ammonia shows the lowest correlation energy but, unlike methane, it is not an outlier because it is only 2.6 SDs away from the average value of 187.8 kJ/mol. Hence, ammonia cannot be omitted and thus only one average is reported. The highest energy value of 201.2 kJ/mol occurs in acetamide, which is only 1 SD above the average. While the total range is 47.4 kJ/mol, it more than halves to 22.3 kJ/mol when ammonia is discarded. Compared to the methyl group the hydrogens of the amino group cluster in a more narrow way (4%) than its nitrogen (9%) does.

**Table 4:** The *A-A’* atomic energies (kJ/mol) of the atoms in the NH_2_ group of various compounds calculated using the (15,15) grid. If the hydrogens in a given compound are global-symmetry-related then their values are averaged. The total average value and SD (in brackets) for all the amine groups are given in the bottom row. Method: 2PDM-M/CCSD(T)/uncontracted 6–31 + G(d, p). The sum in the utmost right column is a sum of the absolute differences with respect to the reference compound CH_3_NH_2_

Molecule	*N*	H1	H2	sum	*N*	H1	H2	sum
NH_3_	104.5	24.6	24.6	153.8	−18.7	−3.2	−3.2	25.1
*CH*_*3*_*NH*_*2*_ *(reference molecule)*	*123.2*	*27.8*	*27.8*	*178.9*	*0.0*	*0.0*	*0.0*	*0.0*
C_2_H_5_NH_2_	126.0	28.8	28.2	183.0	2.8	0.9	0.4	4.1
C_3_H_7_NH_2_	127.3	29.1	28.5	184.9	4.1	1.3	0.7	6.0
*iso*-C_3_H_7_NH_2_	128.3	29.2	29.2	186.6	5.1	1.3	1.3	7.7
HCONH_2_	145.3	27.8	27.0	200.0	22.1	0.0	−0.9	23.0
CH_3_CONH_2_	145.2	28.3	27.6	201.2	22.0	0.5	−0.2	22.7
HN = C(H)NH_2_	142.3	28.2	27.3	197.8	19.1	0.3	−0.5	19.9
NH_2_CH_2_CH_2_OH	128.5	28.4	28.4	185.3	5.3	0.5	0.5	6.3
(CH_3_-NH)C(NH_2_)NH_2_^+^ 1st	141.7	26.6	26.0	194.4	18.5	−1.2	−1.8	21.6
(CH_3_-NH)C(NH_2_)NH_2_^+^ 2nd	141.6	26.7	26.5	194.8	18.4	−1.1	−1.3	20.8
C(NH_2_)_3_^+^	140.7	26.0	26.0	192.8	17.5	−1.8	−1.8	21.1
Average (SD)	132.9 (11.6)	27.6 (1.3)	27.3 (1.2)	187.8 (12.3)	12.2 (7.9)	0.1 (1.0)	−0.3 (0.9)	13.9 (8.2)

### The hydroxyl group

Table 5 presents the results for the OH group as it appears in various hydroxyl-containing compounds. Water has the lowest energy hydroxyl group and thereby stretches the range of the whole set to 53.6 kJ/mol. However, water is less than 3SDs away from the average of 181.1 kJ/mol and hence it is not an outlier. The ratio of the SD to the average is now ~ 7% and carried more or less evenly between O and H. Looking at the energy differences with methanol it is clear that the presence of a C = O group next to OH (i.e. carboxylic acid group) creates the largest disruption to its energy values (25 to 26 kJ/mol).

**Table 5:** The *A-A’* atomic energies (kJ/mol) of the atoms in the OH group in various hydroxyl-containing compounds calculated with the (15,15) grid. The average values are given in the last row, with the SD in brackets. Method: 2PDM-M/CCSD(T)/uncontracted 6–31 + G(d, p). The sum in the utmost right column is a sum of the absolute differences with respect to the reference molecule methanol

Molecule	O	H	sum	O	H	sum
H_2_O	128.3	19.8	148.1	−24.4	−3.1	27.5
*CH*_*3*_*OH (reference molecule)*	*152.7*	*22.9*	*175.6*	*0.0*	*0.0*	*0.0*
C_2_H_5_OH	156.0	23.9	179.8	3.3	0.9	4.2
*n*-C_3_H_7_OH	157.5	24.2	181.6	4.8	1.3	6.0
*iso*-C_3_H_7_OH	159.6	24.2	183.8	6.9	1.3	8.2
NH_2_CH_2_CH_2_OH	158.3	24.1	182.4	5.6	1.2	6.8
HCO_2_H	177.6	22.5	200.1	24.9	−0.4	25.3
CH_3_CO_2_H	178.4	23.3	201.7	25.7	0.4	26.1
(HC = O)CH_2_OH	162.8	22.1	184.9	10.1	−0.8	10.9
CH_3_**OH**^…^O(H)CH_3_	157.5	19.7	177.2	4.8	−3.2	8.1
CH_3_OH^…^**O(H)**CH_3_	154.1	23.2	177.3	1.4	0.3	1.7
Average (SD) include H_2_O	158.4 (12.6)	22.7 (1.5)	181.1 (13.4)	5.7 (12.6)	−0.2 (1.5)	11.3 (9.6)

### Complexes containing water

Because of its utmost importance we next turn our attention to water to find out how it behaves in various environments. Table 6 displays the atomic energies of oxygen and the two hydrogens, both “as is”, and as a difference against the reference of an isolated water molecule. The free water is less than one SD away from the set’s average of 172.4 kJ/mol. Again we ask ourselves how narrow the respective energy distributions are and find that the SD-to-average ratio is 4%, 5–6% and 3% for O, H and H_2_O, respectively. We conclude that water preserves its energetic identity quite well “as seen through the eyes” of electron correlation. However, the singly-charged cationic trimer and tetramer show large differences with the single neutral water (~ 25 kJ/mol, which is the largest seen in Table 6) while the vicinity of a singly negatively charged formate ion also cranks up the difference (to ~ 19 kJ/mol). As expected, in the methane…water complex the energy difference is very small (the smallest in Table 6) and only 1.7 kJ/mol, thereby confirming that the isolated water is preserved to a very large degree. We note that this small energy is stable with respect to grid variation (i.e. (10,10)).

**Table 6:** The *A-A’* atomic energy (kJ/mol) terms of water in various environments. Grid is (15,15). The average value is given in the last row with the SD in brackets. Method: 2PDM-M/CCSD(T)/uncontracted 6–31 + G(d, p). Figure 1 displays the first and second solvating waters of protonated methylamine and the formate ion

Molecule	O	H1	H2	sum	O	H1	H2	sum
*H*_*2*_ *O (reference molecule)*	*128.3*	*19.8*	*19.8*	*167.9*	*0.0*	*0.0*	*0.0*	*0.0*
**H**_**2**_**O**.HOH (acceptor)	130.7	19.9	19.9	170.5	2.4	0.1	0.1	2.6
H_2_O.**HOH** (donor)	132.3	20.5	17.5	170.3	4.1	0.7	−2.3	7.1
H_2_O.**H**_**2**_**O**.H_2_O^a^ Middle H_2_O	132.9	20.0	20.0	172.9	4.6	0.2	0.2	5.0
**H**_**2**_**O**.H_2_O.**H**_**2**_**O**^**a**^ Terminal H_2_O (x2)	132.3	20.5	17.8	170.6	4.0	0.7	−2.0	6.8
NH_3_.H_2_O	134.1	21.0	17.3	172.4	5.8	1.2	−2.5	9.5
NH_4_^+^.H_2_O	130.3	19.0	19.0	168.4	2.1	−0.8	−0.8	3.7
CH_3_NH_3_^+^.H_2_O	130.6	19.4	19.4	169.3	2.3	−0.4	−0.4	3.2
CH_3_NH_3_^+^.(H_2_O)_2_ Fig. 1 (1)	130.4	19.5	19.5	169.5	2.2	−0.9	−0.9	3.9
CH_3_NH_3_^+^.(H_2_O)_2_ Fig. 1 (2)	130.4	19.5	19.5	169.5	2.2	−2.4	−0.3	4.9
HCO_2_^−^.H_2_O	145.1	19.0	19.5	183.5	16.8	0.1	−0.3	17.2
HCO_2_^−^.(H_2_O)_2_ Fig. 1 (1)	145.4	17.4	21.5	184.4	17.2	0.3	1.7	19.2
HCO_2_^−^.(H_2_O)_2_ Fig. 1 (2)	143.0	19.9	17.7	180.7	14.8	−2.1	−2.1	19.0
CH_4_.H_2_O	128.7	20.1	20.1	168.9	0.4	−1.0	0.3	1.7
H_5_O_2_^+^	136.4	17.7	17.6	171.7	8.1	−0.8	−2.2	11.0
H_7_O_3_^+^	132.6	18.8	18.7	170.0	4.3	−19.8	−1.1	25.2
H_9_O_4_^+^	132.1	19.1	19.2	170.3	3.8	−19.8	−0.7	24.3
Average (SD) include H_2_O	133.9 (5.3)	19.5 (0.9)	19.1 (1.1)	172.4 (5.1)	5.6 (5.3)	−0.3 (0.9)	−0.8 (1.1)	7.3 (6.1)

^a^ The water trimer was forced to be a linear geometry (i.e open structure) rather than a cyclic one, as is found in the minimum energy form

### The carbonyl group

So far we have considered saturated bonding situations but here, for the first time, we consider double bonds starting with the carbonyl group. Table 7 lists the results. Before analysing the energies a technical point on the grid size needs to be mentioned. There is a need for a larger grid than that used for the saturated carbon (i.e. the CH_3_ group). We added the formate and acetate ions to be compared to formic acid and acetic acid, respectively. The differences between these two pairs (i.e. ion and neutral acid) are smaller than the differences between formic acid and acetic acid (Table 7).

**Table 7:** The *A-A’* atomic energy terms of C = O in various chemical environments (kJ/mol). The average values are given in the last two rows, with the standard deviation in brackets. Method: 2PDM-M/CCSD(T)/uncontracted 6–31 + G(d, p). Grid (15,15). The sum is a sum of the absolute energies. The final three columns on the right give the energies relative to those of acetaldehyde and of acetone (in brackets)

Molecule	C	O	sum	C	O	sum
CH_2_O	122.6	194.0	316.6	−8.2 (−16.0)	−2.8 (−6.5)	11.0 (22.5)
*CH*_*3*_*CHO* *(reference molecule)*	*130.8*	*196.8*	*327.6*	*0.0 (−7.7)*	*0.0 (−3.7)*	*0.0 (11.5)*
C_2_H_5_CHO	132.2	196.7	328.9	1.4 (−6.4)	−0.1 (−3.9)	1.5 (10.2)
n-C_3_H_7_CHO	132.2	197.3	329.5	1.3 (−6.4)	0.5 (−3.2)	1.8 (9.6)
iso-C_3_H_7_CHO	133.0	198.2	331.2	2.1 (−5.6)	1.4 (−2.3)	3.6 (7.9)
*CH*_*3*_*C(O)CH*_*3*_ *(2nd ref. molecule)*	*138.6*	*200.5*	*339.1*	*7.7 (0.0)*	*3.7 (0.0)*	*11.5 (0.0)*
CH_3_C(O)C_2_H_5_	139.2	199.6	338.9	8.4 (0.6)	2.9 (−0.9)	11.3 (1.5)
HC(O)NH_2_	123.9	189.6	313.5	−6.9 (−14.7)	−7.2 (−10.9)	14.1 (25.6)
CH_3_C(O)NH_2_	131.6	193.3	324.9	0.8 (−6.9)	−3.5 (−7.2)	4.3 (14.2)
HC(O)N(H)CH_3_	126.8	191.2	317.9	−4.1 (−11.8)	−5.6 (−9.3)	9.7 (21.2)
CH_3_C(O)N(H)CH_3_	134.1	194.1	328.2	3.3 (−4.5)	−2.7 (−6.4)	5.9 (10.9)
HCO_2_H	126.9	193.0	319.9	−3.9 (−11.7)	−3.8 (−7.5)	7.8 (19.2)
HCO_2_^−^	130.2	192.8	323.0	−0.6 (−8.3)	−4.0 (−7.8)	4.6 (16.1)
CH_3_CO_2_H	135.7	196.9	332.7	4.9 (−2.9)	0.1 (−3.6)	5.0 (6.4)
CH_3_CO_2_^−^ (conf. A)^a^	134.7	194.7	329.4	3.8 (−3.9)	−2.1 (−5.9)	6.0 (9.8)
CH_3_CO_2_^−^ (conf. B)	135.5	194.7	330.1	4.6 (−3.1)	−2.1 (−5.8)	6.7 (9.0)
CHOCH_2_OH	133.7	196.7	330.4	2.8 (−4.9)	0.0 (−3.8)	2.9 (8.7)
Average (SD)	131.9 (4.5)	195.3 (2.8)	327.2 (6.9)	1.6 (4.0)	−1.4 (2.9)	6.0 (3.8)

^a^For acetate we consider two conformations: *A* with the unique methylic hydrogen in the CO_2_plane, and *B* with this hydrogen at right angles to the CO_2_plane, both with C_s_ symmetry. For conformation A, the different C-O lengths have been averaged

The average correlation energy for a carbonyl group is 327.2 kJ/mol with a SD of 6.9 kJ/mol. The latter is the same as that for the methyl group but because the average is almost twice (1.7) as large, the ratio SD-to-average drops from 4% to 2%. Hence, the energy distribution is much sharper, that is, more centred around its average value. Furthermore, the energy distribution of oxygen is about 2.5 times sharper than that of carbon. The range is also much smaller (about half) compared to that of the methyl: 26 kJ/mol based on the minimum value of 313.5 kJ/mol (formamide) and the maximum value of 339.1 kJ/mol (acetone). Neither extreme is an outlier so no data points should be excluded from the average. Finally, this time acetaldehyde is introduced as the reference molecule, which generating the difference energies in the utmost right columns of Table 7. It turns out that formamide generates the largest difference, caused by substituting the methyl group by an amino group. However, if the opposite is done, that is substituting the hydrogen in acetaldehyde by an amino group, then one of the smaller energy differences (4.3 kJ/mol) in the Table appears. In line with chemical intuition, substituting the methyl for an ethyl creates a very small difference: 1.5 kJ/mol.

### The carboxyl group

In previous sections we discussed the OH and C = O groups; both are part of the carboxylic acid group, which is discussed here as a single entity. We focus on a small series of molecules, denoted GCOOH where G = H, CH_3_ or C_2_H_5_, alongside their respective anions, both unsolvated (gas phase) and hydrated (with one or two water molecules). Table 8 shows the by now familiar column organisation but with the extra feature that now two reference molecules have been introduced, one for the neutral acid and one for the anion: HCO_2_H and HCO_2_^−^. A simple observation to make in Table 8 is that the carboxylic hydrogen (when it occurs) is sharply defined because of its narrow range averaging 23.1 kJ/mol. Secondly, both in the neutral and anionic state, the correlation energy increases from H to CH_3_, and from CH_3_ to C_2_H_5_, first by 9–14 kJ/mol and then by only 1–2 kJ/mol. While these two series are too short to prove asymptotic convergence, the hypothesis is viable. Neither the neutral or anionic compounds show outliers by the typical 3SD criterion. The anionic average is unusually sharp by its SD-to-average ratio of 1%, which is not due to the SD value itself (which is the typical 5–13 kJ/mol seen for methyl, amino, hydroxyl or carboxy groups) but by the relatively large average value itself. We note there is a small conformational energy difference for the two conformers of acetate. Finally, hydration (solvation) lowers the correlation energy in the formate anion upon each addition of a water but not so for the acetate.

**Table 8:** The *A-A’* atomic energy terms of CO_2_H and CO_2_^−^ groups in various chemical environments (kJ/mol). The average values are given in the last row for the anions only, with the standard deviation in brackets. Method: 2PDM-M/CCSD(T)/uncontracted 6–31 + G(d, p). Grid is (15,15)

Molecule	C	O(= C)	O(-H) or O^−^(-C)	H	sum	C	O(= C)	O(-H) or O^−^(-C)	H	sum
*HCO*_*2*_*H (reference)*	*126.9*	*193.0*	*177.6*	*22.5*	*520.0*	*0.0*	*0.0*	*0.0*	*0.0*	*0.0*
*HCO*_*2*_^*−*^ *(reference)*	*130.2*	*192.8*	*192.8*		*515.8*	*0.0*	*0.0*	*0.0*		*0.0*
HCO_2_^−^. H_2_O	129.0	192.2	192.2		513.4	−1.2	−0.6	−0.6		−2.3
HCO_2_^−^.(H_2_O)_2_	127.0	190.0	191.3		508.3	−3.3	−2.7	−1.5		−7.5
CH_3_CO_2_H	135.7	196.9	178.4	23.3	534.4	8.8	3.9	0.8	0.8	14.3
CH_3_CO_2_^−^ (conf. A)	134.7	195.3	194.0		524.0	4.4	2.5	1.3		8.3
CH_3_CO_2_^−^ (conf. B)	135.5	194.7	194.7		524.8	5.2	1.9	1.9		9.0
CH_3_CO_2_^−^.H_2_O	138.4	194.9	193.8		527.1	8.1	2.2	1.1		11.4
C_2_H_5_CO_2_H	136.9	197.0	179.2	23.6	536.7	10.0	4.0	1.5	1.1	16.6
C_2_H_5_CO_2_^−^	136.0	195.0	194.1		525.1	5.7	2.3	1.3		9.3
Average (SD) anions	133.0 (3.9)	193.6 (1.8)	193.3 (1.1)		519.8 (6.7)	3.2 (4.0)	0.9 (1.9)	0.6 (1.2)		4.7 (7.0)

### The amide group

Here we bring together in one place data already given in two previous tables. Table 9 presents the atomic energetics of the amide group, which consists of energies of the carboxyl and the amino group combined. There are four molecules, starting with formamide, which upon substitution of one hydrogen by one methyl leads to either acetamide or N-methylformamide depending on which hydrogen was replaced. Finally, the substitution of two hydrogens results in N-methylacetamide, the famous “NMA” molecule that often performs as the smallest representative of a peptide bond in proteins. This molecule is taken as the reference to calculate the energy differences listed in the right half of Table 9. The entries can be explained by the systematic substitution pattern mentioned just above. The oxygen acts as a spectator because it is not directly affected by a methyl substitution through a change in bonding to itself. This is why its electron correlation energies vary less than those of C and N, whose bonding is indeed altered. Finally, looking at the “sum” column in the middle of Table 9 confirms that the concept of the amide functional group survives with an average value of 515.4 kJ/mol and a SD of 7.4, which corresponds to a very sharp SD-to-average ratio of only 1%.

**Table 9:** The *A-A’* atomic energy terms of amide groups in various chemical environments (kJ/mol). Method: 2PDM-M/CCSD(T)/uncontracted 6–31 + G(d, p). Grid is (15,15). The last six columns give the energetic relative to N-methylacetamide

Molecule	C	O	*N*	H	H	sum	C	O	*N*	H	H	sum
HCONH_2_	123.9	189.6	145.3	27.8	27.0	513.6	−10.2	−4.5	−12.5	−2.8	27.0	−3.0
CH_3_CONH_2_	131.6	193.3	145.2	28.3	27.6	526.1	−2.4	−0.8	−12.6	−2.3	27.6	9.5
HCON(H)CH_3_	126.8	191.2	157.4	30.2		505.4	−7.3	−2.9	−0.5	−0.4	0.0	−11.1
CH_3_CON(H)CH_3_	134.1	194.1	157.8	30.6		516.6	0.0	0.0	0.0	0.0	0.0	0.0
Average (SD)	129.1 (4.0)	192.0 (1.8)	151.4 (6.2)	29.2 (1.2)		515.4 (7.4)						

### The CH_2_ group

Table 10 shows the energetics of the CH_2_ group and the energy differences with “asym” propane as the reference molecule. As before with other functional groups, the methylene group also expresses itself sharply (4%) around its own average value, which is 174.2 kJ/mol with a typical SD of ~ 6 kJ/mol. The range, from 164.0 to 186.2 kJ/mol for propane and formaldehyde, respectively, amounts to 22.2 kJ/mol. By the usual 3SD criterion both the minimum and maximum are outliers and should thus be excluded from the calculation of the average, in order to keep this analysis consistent with that of other functional groups. However, the bottom row of Table 10 shows that this correction does not matter much.

**Table 10:** The *A-A’* atomic energy terms of CH_2_ groups in various environments (kJ/mol). Method: 2PDM-M/CCSD(T)/uncontracted 6–31 + G(d, p). Grid is (15,15)

Molecule	C	H	H	sum	C	H	H	sum
*C*_*3*_*H*_*8*_ *(asym)*	*99.1*	*32.5*	*32.5*	*164.0*	*0.0*	*0.0*	*0.0*	*0.0*
C_3_H_8_ (sym)	99.6	32.5	32.5	164.5	0.5	0.0	0.0	0.5
C_4_H_10_	101.4	32.7	32.7	166.8	2.3	0.2	0.2	2.8
C_2_H_5_NH_2_	102.0	32.7	33.2	167.9	3.0	0.2	0.7	3.9
CH_3_CH_2_**CH**_**2**_NH_2_	104.1	32.9	33.4	170.4	5.1	0.5	0.9	6.4
CH_3_**CH**_**2**_CH_2_NH_2_	101.5	32.7	32.8	167.0	2.4	0.2	0.4	3.0
C_2_H_5_OH	104.6	33.1	33.1	170.7	5.6	0.6	0.6	6.8
CH_3_CH_2_**CH**_**2**_OH	106.7	33.3	33.3	173.3	7.7	0.8	0.8	9.3
CH_3_**CH**_**2**_CH_2_OH	100.8	32.5	32.5	165.8	1.7	0.1	0.1	1.8
CH_2_O	122.6	31.8	31.8	186.2	23.5	−0.7	−0.7	22.2
C_2_H_5_CHO	106.6	34.0	34.0	174.7	7.6	1.6	1.6	10.7
CH_3_**CH**_**2**_CH_2_CHO	101.8	33.0	33.0	167.9	2.8	0.6	0.6	3.9
CH_3_CH_2_**CH**_**2**_CHO	108.0	34.2	34.2	176.3	8.9	1.7	1.7	12.4
C_2_H_4_	117.8	32.1	32.1	182.0	18.8	−0.4	−0.4	18.0
CH_2_NH	119.6	32.3	32.8	184.7	20.5	−0.1	0.3	20.7
C_2_H_5_OCH_3_	106.9	33.3	33.3	173.5	7.8	0.9	0.9	9.5
C_2_H_5_CO_2_^−^	111.3	35.5	36.1	182.8	12.2	3.0	3.6	18.9
C_2_H_5_CO_2_H	106.8	34.1	34.1	175.0	7.8	1.6	1.6	11.0
C_2_H_5_C ≡ CH	107.3	34.5	34.5	176.3	8.2	2.1	2.1	12.3
CH_3_**CH**_**2**_CH_2_C ≡ CH	103.4	34.4	33.6	171.4	4.4	2.0	1.1	7.4
CH_3_CH_2_**CH**_**2**_C ≡ CH	111.0	35.4	34.8	181.2	11.9	3.0	2.3	17.2
CH_3_C ≡ CC_2_H_5_	107.7	34.8	34.8	177.4	8.7	2.4	2.4	13.4
CH_3_COC_2_H_5_	107.9	34.4	34.2	176.5	8.8	2.0	1.7	12.5
CHOCH_2_OH	112.5	34.5	34.5	181.6	13.4	2.1	2.1	17.6
CH_3_C(H)NC_2_H_5_	109.3	35.1	34.1	178.4	10.2	2.6	1.6	14.4
Average (SD)	107.2 (6.0)	33.5 (1.1)	33.5 (1.0)	174.2 (6.4)	8.5 (5.8)	1.1 (1.1)	1.1 (1.0)	10.7 (6.2)
Average (SD) exclude CH_2_O and C_3_H_8_	107.2 (4.9)	33.7 (1.0)	33.7 (0.9)	174.6 (5.6)	8.2 (4.9)	1.3 (1.0)	1.2 (0.9)	10.6 (5.6)

### The triple bond

In a broader context the triple bond is of interest for three reasons. Firstly, there is an enzyme that hydrates the ethyne (or acetylene) molecule called acetylene hydratase [27]– [28]. Secondly, molecular nitrogen[29], the nitrosium ion^[30]^ (NO^+^), the well-known toxic species the cyanide ion [31] and carbon monoxide [32], are all of biological significance and triple-bonded. Thirdly, by employing the CCSD(T) approach, this method simultaneously excites electrons from all three bonds. In theory, this last point allows a description of dissociation but without orbital optimisation, the description is unlikely to be perfect.

### The alkynes

Various substituted alkynes suffer from linear dependency in the basis set and therefore we have removed from the basis set of carbon the outermost *s*-function, which is part of an *sp* set of functions. Table 11 reports the atomic energetics. The hydrogens attached to a CC triple bond have a narrow energy distribution with a SD-to-average ratio of almost 3% while that of the triple-bonded carbons is about 4%.

**Table 11:** The *A-A’* energetics of the acetylenic triple bond in various alkynes. Note that the sum only includes the two carbons. The method is 2PDM-M/CCSD(T) and the basis set is uncontracted 6–31 + G(d, p) with the outer *s*-function deleted from the carbon basis set. The C2 atom is marked as bold in the chemical formulae. Units: kJ/mol

Molecule/atom	C1	C2	H	sum
H**C** ≡ **C**H	134.7	134.7	33.4	269.4
CH_3_C ≡ **C**H	144.6	136.7	34.6	281.3
CH_3_**C** ≡ **C**CH_3_	147.2	147.2		294.4
C_2_H_5_C ≡ **C**H	145.3	138.0	34.9	283.6
C_2_H_5_**C** ≡ CCH_3_	147.7	147.2		294.8
C_3_H_7_C ≡ **C**H	144.4	138.3	35.1	282.6
HC ≡ **C**-**C** ≡ CH	148.8	158.4	35.9	307.2
Average (SD)	143.4 (5.2)	141.9 (7.8)	34.5 (0.9)	285.3 (12.2)

To analyse the deeper significance of the triple excitations, we employed a somewhat larger basis set on acetylene [uncontracted 6–31 + + G(2d,2p)] and looked at its overall energetics in detail. In addition, we employed three approaches, Hartree-Fock, CCSD and CCSD(T). Table 12 gives the results. As expected, the effect of electron correlation is significant and of the order of hundreds of kJ/mol, as shown in the final two columns of Table 12. Secondly, perhaps somewhat unexpectedly, the effects of perturbative triples can be moderately large compared to CCSD alone, with the *ee* Coulomb and nuclear attraction energies differing by over 100 kJ mol^− 1^ between CCSD and CCSD(T). The effects of triple excitations on the underlying CCSD wavefunction is to increase the relevant one-electron terms but decrease two-electron energetic terms, at least in the Müller approximation.

**Table 12:** Charges and energetics of the Hartree-Fock, CCSD and CCSD(T) wavefunctions of ethyne (basis set uncontracted 6–31 + + G(2d,2p)) for a single carbon atom. Units: atomic units of energy and of energy, and kJ/mol for energy differences

Property/Method	Hartree-Fock	CCSD	CCSD(T)	CCSD-HF	CCSD(T)-HF
Atomic Charge	−0.101	−0.100	−0.094	0.001	0.007
Kinetic	37.750963	37.973340	37.973907	583.9	585.3
Nuclear Attraction	−108.851238	−109.083052	−109.037630	−608.6	−489.4
Müller ee Coulomb	28.284483	28.419878	28.382828	355.5	258.2
Müller ee Exchange	−5.167244	−5.521370	−5.539321	−929.8	−976.9

#### Triple-bonded diatomic molecules

The diatomic molecules N_2_, CO and CN^−^ are perhaps the best-known triple bonded systems, although NO^+^ should be better known, given its importance in the biological sphere. Table 13 gives the energetic breakdown of these systems employing the Hartree-Fock, CCSD and CCSD(T) methods, with the uncontracted 6–31 + + G(2d,2p) basis sets. As for ethyne, the values generally show similar CCSD and CCSD(T) values, with the Hartree-Fock values being somewhat different. The effect of the triple excitations (CCSD(T)), on top of the CCSD wavefunctions, is to decrease the exchange energy (Müller approximation) and generally decrease the Coulomb term (Müller approximation) but the oxygen of NO^+^ is the only exception to this rule. The nuclear attraction energy generally increases with the addition of triples but again the oxygen of the nitrosonium ion shows a decrease. Kinetic energy both increases and decreases upon inclusion of the perturbative triple excitations.

**Table 13:** The atomic charges and energy components for N2, CO, CN- and NO+. The two energy rows each time correspond to the atoms in the order given by the chemical formula. The basis set is the uncontracted 6–31++G(2d,2p). Units: atomic units of charge and of energy

**Energetics**	**N**_**2**_	**CO**	**CN**^**-**^	**NO**^**+**^
Atomic Charge				
Hartree-Fock	0.000	1.348	0.759	1.420
	0.000	−1.348	−1.759	−0.420
CCSD	0.00	1.172	0.521	1.168
	0.00	−1.172	−1.521	−0.168
CCSD(T)	0.00	1.166	0.517	1.196
	0.00	−1.166	−1.517	−0.196
Kinetic				
Hartree-Fock	54.329873	36.794722	36.973937	53.255352
	54.329873	75.724775	55.159294	75.276037
CCSD	54.637690	37.074539	37.300041	53.800982
	54.637690	75.934882	55.329353	75.436990
CCSD(T)	54.630614	37.066808	37.296669	53.744685
	54.630614	75.957274	55.334760	75.487882
Nuclear Attraction				
Hartree-Fock	−151.337955	−104.149828	−103.946056	−146.770194
	−151.337955	−206.339317	−154.281213	−206.234648
CCSD	−151.700449	−105.101208	−104.979003	−148.608848
	−151.700449	−205.813367	−153.714585	−205.395267
CCSD(T)	−151.647213	−105.079625	−104.943228	−148.377919
	−151.647213	−205.795241	−153.653548	−205.553988
Müller ee Coulomb				
Hartree-Fock	37.296918	22.997035	24.302869	31.637980
	37.296918	53.090958	42.491510	50.544877
CCSD	37.473054	23.763683	25.207832	33.014069
	37.473054	52.453505	41.785519	49.598243
CCSD(T)	37.439412	23.759773	25.187969	32.842681
	37.439412	52.429036	41.731036	49.731274
Müller ee Exchange				
Hartree-Fock	−6.543091	−4.562502	−4.708048	−5.906270
	−6.543091	−8.750759	−7.157649	−8.437986
CCSD	−6.986997	−4.901400	−5.103511	−6.427841
	−6.986997	−9.235094	−7.572119	−8.856932
CCSD(T)	−7.010745	−4.921212	−5.125422	−6.435881
	−7.010745	−9.263110	−7.599368	−8.902611

## Geometric dependence of the correlation energy

### Conformational dependence of the energetics (dihedral angle rotation)

We reiterate that the energetics given in the Tables 14, 15, 16, 17, 18, 19, and 20, which is our work on conformational energetics, were obtained at the 2PDM-M/CCSD(T)/uncontracted 6–31 + G(d, p) level but the geometries were optimised at the CCSD(FULL)/uncontracted 6–31 + G(d, p) level.

**Table 14:** Conformational energetics of ethane’s atoms. Method: 2PDM-M/CCSD(T)/uncontracted 6–31 + G(d, p). Grid is (15,15). Units: kJ/mol

atom	Minimum	Optimised eclipsed	Rigid rotation eclipsed
C1	87.2	87.4	87.1
C2	87.2	87.4	87.1
H3	29.9	29.8	29.8
H4	29.9	29.8	29.8
H5	29.9	29.8	29.8
H6	29.9	29.8	29.8
H7	29.9	29.8	29.8
H8	29.9	29.8	29.8

**Table 15:** Propane’s atoms conformational dependence of their two-electron energies. Method: 2PDM-M/CCSD(T)/uncontracted 6–31 + G(d, p). Grid is (15,15). Units: kJ/mol. The description of the atoms refers to the 120° conformation

Atom/Angle	120° (eclipsed)	140°	160°	180°	Average	SD
C1 (staggered)	89.6	89.6	89.8	89.8	89.7	0.1
C2 (CH_2_)	99.1	99.0	99.0	99.5	99.1	0.2
C3 (rotated)	90.4	90.3	90.1	89.8	90.1	0.2
H4 (-C2, eclipse H9)	32.5	32.5	32.5	32.5	32.5	0.0
H5 (-C2, eclipse H10)	32.5	32.5	32.5	32.5	32.5	0.0
H6 (-C1)	30.4	30.4	30.3	30.3	30.3	0.0
H7 (-C1, in plane)	31.2	31.2	31.1	31.1	31.1	0.0
H8 (-C1)	30.4	30.4	30.3	30.3	30.3	0.0
H9 (-C3, eclipse H4)	30.7	30.9	31.1	31.1	31.0	0.1
H10 (-C3, eclipse H5)	30.7	30.6	30.4	30.3	30.5	0.1
H11 (-C3, eclipse C1)	30.1	30.1	30.2	30.3	30.2	0.1

**Table 16:** The atomic energies for methylamine in various conformations. Grid is (15,15). One dihedral angle is fixed while the other geometric parameters have been optimised. The atom labels refer to the eclipsed conformation. Method: 2PDM-M/CCSD(T)/uncontracted 6–31 + G(d, p). Unit: kJ/mol

Atom/conformation	eclipsed	20.0	40.0	minimum	average	SD
N1	122.3	122.0	122.5	123.2	122.5	0.4
C2	91.0	90.7	90.7	90.6	90.8	0.1
H3 (-C, eclipse H6)	30.1	29.9	29.9	30.0	30.0	0.1
H4 (-C, eclipse H7)	30.1	30.4	30.6	30.7	30.5	0.2
H5 (-C)	30.4	30.3	30.2	30.0	30.2	0.2
H6 (-N)	27.7	27.6	27.7	27.8	27.7	0.1
H7 (-N)	27.7	27.7	27.8	27.8	27.7	0.1

**Table 17:** The atomic energies of methanol in various conformations. One dihedral angle is fixed and the other geometric parameters were optimised. The atom labels refer to the eclipsed conformation. Grid is (15,15). Method: 2PDM-M/CCSD(T)/uncontracted 6–31 + G(d, p). Unit: kJ/mol

Atom/conformation	eclipsed	20.0	40.0	minimum	average	SD
C1	93.0	93.0	93.0	92.9	93.0	0.1
O2	152.3	152.4	152.6	152.7	152.5	0.2
H3 (-O, eclipse H4)	22.8	22.8	22.9	22.9	22.8	0.1
H4 (-C, eclipse H3)	30.0	30.1	30.2	30.3	30.2	0.1
H5 (-C)	30.0	30.2	30.3	30.3	30.2	0.1
H6 (-C)	30.0	29.8	29.6	29.5	29.7	0.2

**Table 18:** The data from Table 8 for the acid group of the acetate ion. The Methyl group has a hydrogen in the CO_2_ plane, for conf. A, and at right angles to CO_2_ for conf. B. Method: 2PDM-M/CCSD(T)/uncontracted 6–31 + G(d, p). Grid is (15,15). Unit: kJ/mol

atoms	C	O1	O2	sum
CH_3_CO_2_^−^ (conf. A)	4.4	2.5	1.3	8.3
CH_3_CO_2_^−^ (conf. B)	5.2	1.9	1.9	9.0

**Table 19:** The corresponding atomic energies of the Methyl group for the conformations of the acetate ion given in Table 18. H2 has a symmetry-related atom, which is not given. Method: 2PDM-M/CCSD(T)/uncontracted 6–31 + G(d, p). Grid is (15,15). Unit: kJ/mol

atoms	C	H1 (in plane)	H2 (out of plane)	sum
CH_3_CO_2_^−^ (conf. A)	101.2	33.2	33.6	168.1
CH_3_CO_2_^−^ (conf. B)	101.4	33.8	33.3	168.5

**Table 20:** The atomic energies of ethanol in various conformations. One dihedral angle is fixed and the other geometric parameters optimised. The atom labels refer to the eclipsed conformation. Grid is (15,15). Note here we eclipse along the C-C bond, while in Table 17 (methanol) we eclipsed along the C-O bond. Method: 2PDM-M/CCSD(T)/uncontracted 6–31 + G(d, p). Unit: kJ/mol

Atom/conformation	eclipsed	20.0	40.0	minimum	average	SD
C1 (CH_3_)	90.6	90.3	90.1	89.8	90.2	0.3
C2 (CH_2_)	105.2	105.1	104.7	104.6	104.9	0.2
O3 (-C2, eclipse H7)	156.5	156.4	156.0	156.0	156.2	0.2
H4 (-O3, trans to CH_3_)	24.0	24.0	23.9	23.9	23.9	0.1
H5 (-C2)	33.1	33.1	33.1	33.1	33.1	0.0
H6 (-C2)	33.1	33.1	33.1	33.1	33.1	0.0
H7 (-C1, eclipse O)	29.5	29.6	29.9	30.1	29.8	0.2
H8 (-C1, eclipse H5)	30.5	30.7	30.8	30.8	30.7	0.1
H9 (-C1, eclipse H6)	30.5	30.3	30.3	30.1	30.3	0.1

Table 14 displays the effects of rigid rotation from the minimum to the eclipsed form of ethane, as well as the same rotation followed by geometry-optimisation. There is very little effect on the energetics upon conformational change, whether by rigid rotation or by rotation allowing for the relaxation of bond lengths and angles. Table 14 clearly shows energy differences of the order of tenths of kilojoules per mole with a maximum of 0.3 kJ/mol.

Table 15 shows the atomic energetics and their dependence of conformational change in propane induced by dihedral rotation. These values were obtained by fixing the dihedral angle and optimising the rest of the geometric parameters. The SD is clearly very small and of the order of 0.1 kJ/mol. As for ethane, we conclude that the atomic correlation energies depend very little on conformation, only of the order of 0.1 kJ/mol or less.

Table 18 presents again the results already given in Table 8 (columns 7 to 11), for the acetate ion. It is clear there is a rotational dependence on energetics of the oxygen atoms, when referenced to formate anion, although the sum of these in both conformations is the same (3.8 kJ/mol). Carbon also shows a modest variation of less than 1 kJ/mol.

Table 19, which expands the data given in Table 18, indicates that the methyl group has a small conformational effect on the energetics of the acetate ion. The hydrogen values are very similar to each other for both conformations.

Tables 16, 17, and 20 focus on the effect of conformation on the atomic energetics of methylamine, methanol and ethanol, respectively. Again, very small energy changes emerge, of the order of 0.1 kJ/mol, as observed before, in Tables 14, 15, and 18.

### Non-Dihedral geometry dependence of the energetics

**Fig. 2:**
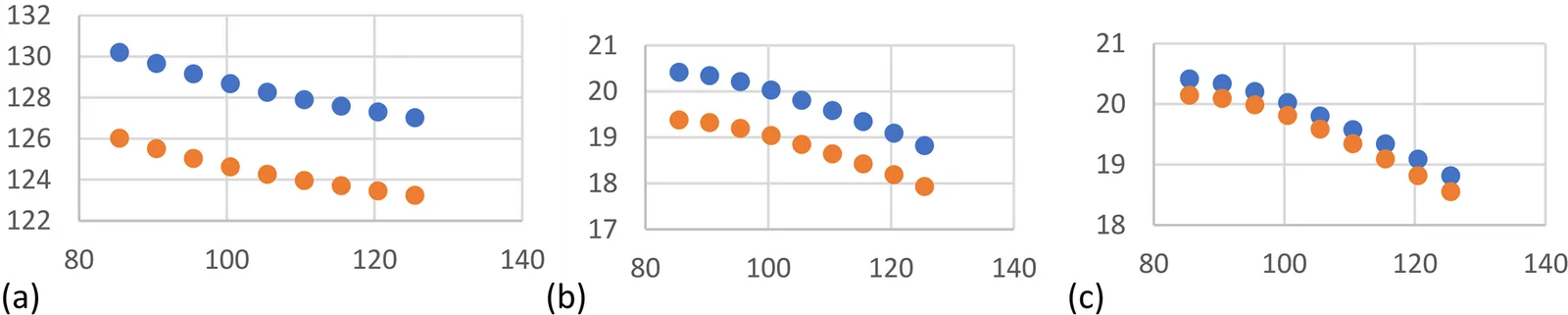
The atomic energy of (a) O, (b) the first H (i.e. H1), and (c) the second H (i.e. H2) in H_2_O for a series of HOH angles (x-axis). The blue profile pertains to the O-H bonds being at their minimum energy bond length values while the orange profile relates to one O-H (H1) bond length being compressed to 0.9 Å (orange). Method 2PDM-M/CCSD(T). Units are degrees (x-axis) and kJ/mol (y-axis)

**Fig. 3:**
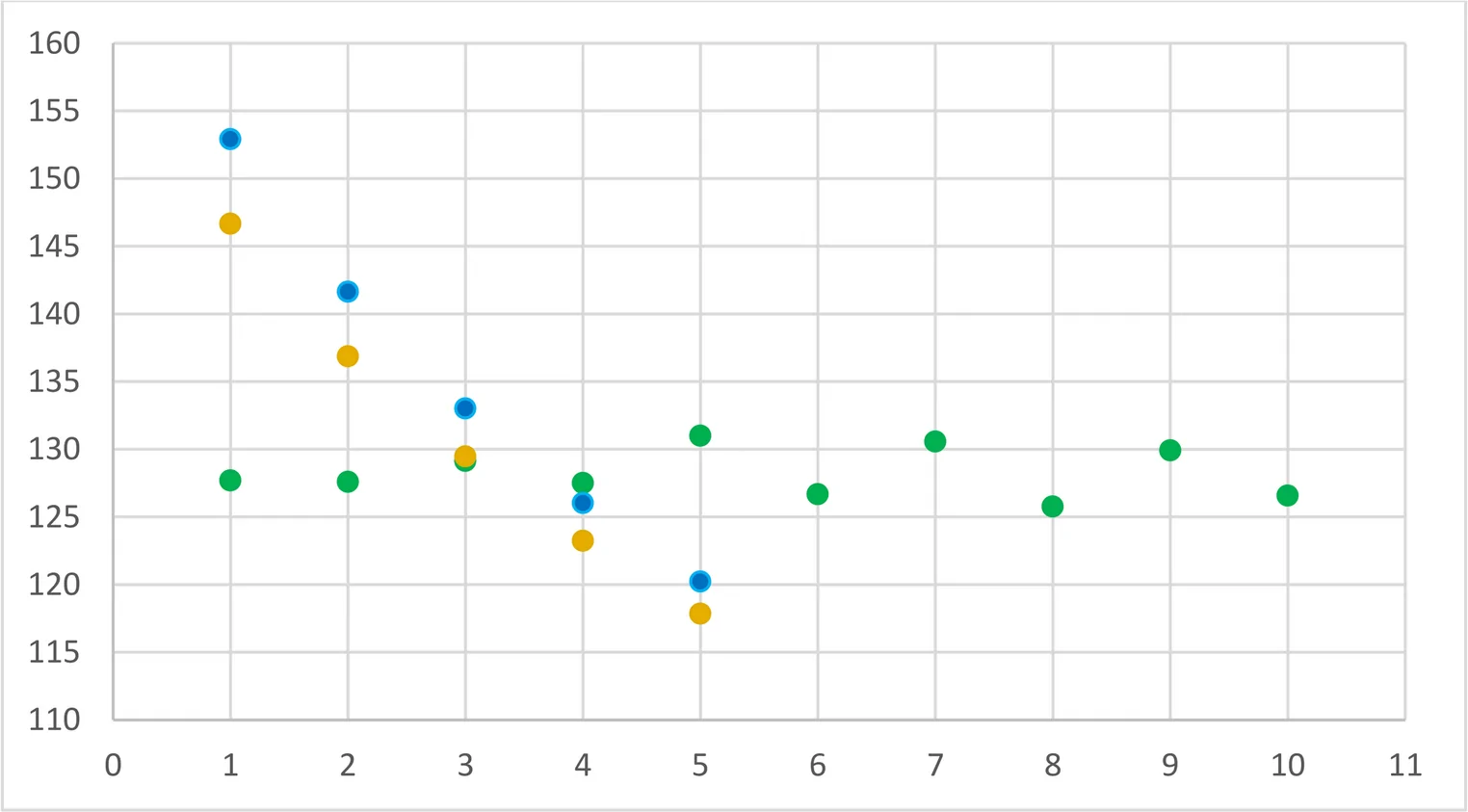
The energetics of the O of H_2_O for 10 random structures (green) compared to the systematic geometry changes (blue and yellow). Method 2PDM-M/CCSD(T). Units are kJ/mol (y-axis) and a number labelling the structure (x-axis). Blue is for a fixed bond angle of 85.5°, yellow for a fixed angle of 125.5°, while one bond length varies from 1.2 to 0.8 Å and the other is held at the optimal value for the minimum (0.962 Å)

Here we briefly consider how correlation energy changes with geometry other than by dihedral rotation. Therefore we looked at changes in bond length and valence angle, as illustrated in a single water molecule. Fig. 2 covers the first type of systematic, manual variation in geometry. However, such variation may be deemed rather artificial. More realistic for a force field in action is a molecular dynamics run where one comes across random changes in geometry. This is why we look at ten geometries randomly taken from such a run. This non-systematic, quasi-random distribution is shown in Fig. 3.

Figure 2 shows that the energetics of the three atoms in water vary smoothly with systematic geometry changes involving the bond angle and one O-H bond. The oxygen follows a nearly straight line. Ideally, one would like a horizontal line, which implies that the response of atomic energetics to geometry variation is the same for both the full 2PDM and the approximate Müller 2PDM.

Figure 3 compares the behaviour of oxygen’s correlation energy for such a run with that induced by systematic changes in geometry, where the bond angles were held fixed once at 85.5° and a second time at 125.5°, while of one of the bond lengths was systematically reduced from 1.2 to 0.8 Å, and the other bond length held fixed at the optimum value (0.962 Å). We see that the molecular dynamics run has a much smaller spread of values than the systematically changed geometries. These values form a horizontal pattern rather than the sloping pattern of the latter. The good news is that ML will be able to cope with any of these variations.

## Conclusions

Quantum topological atoms have been chosen as the basis for the design of a machine learning interatomic potential called FFLUX. All its types of energetic contribution (kinetic, Coulomb, exchange, electron correlation) come from the same energy decomposition scheme called Interacting Quantum Atoms, which embraces the idea of a quantum topological atom. As a result, dispersion effects are seamlessly incorporated in FFLUX, eliminating the need for external dispersion models, no matter how popular.

The work presented in this paper establishes the practical transferability at CCSD(T) level of a good number of functional groups as they appear in small organic molecules: methyl, amine, hydroxyl, water, carbonyl, carboxyl, amide, methylene and a variety of triple bonds. We find that these groups show narrow correlation energy distributions, with a typical standard-deviation-to-average ratio of 1% to 9%, where the standard deviations themselves are at least 5 kJ/mol in magnitude, increasing to 13 kJ/mol for the hydroxyl group. Given it utmost importance, the water “functional group” deserves a special mention. While the ‘electron correlation’ (as a pure two-body effect) of an isolated molecule is 168 kJ/mol, this value always increases when present in any complex (to a maximum of 184 kJ/mol) and an average of 172 kJ/mol, We note that, at CCSD level, the ‘correlation energy’ of a terminal methyl in a series of linear aliphatic chains converged up to 0.1 kJ/mol by the chain length of pentane. Finally, the triple bonds were extensively analysed in terms of the influence of the perturbative triples of CCSD(T) on the underlying CCSD wave function. Surprisingly, their effect is as large as ~ 50–120 kJ/mol for the three types of potential energy but only a few kJ/mol for the kinetic energy.

Conformational dependence in terms of dihedral angles is very small, of the order of tenths of a kJ/mol. However, the dependence of atomic correlation energies of bond length and angle changes in water is of the order of units of a kJ/mol for random changes based on molecular dynamics simulation and tens of kJ/mol for large manually induced geometry changes.

Overall, we are confident that the atomic or functional group energies are sufficiently clustered around numerical averages that are in line with the repeatedly established transferability of quantum topological atoms. We can now add pure electron correlation CCSD(T) to the list of transferable atomic properties. Hence, the machine learning of these chemically transferable energies fits within the strategy of the force field FFLUX, which is thus en route to have its very own representation of dispersion effects.

## Data Availability

The data that supports the findings of this study are available from the corresponding author upon request.
